# Environmental sustainability and food provision in the early childhood and education setting

**DOI:** 10.1017/S1368980023001908

**Published:** 2023-11

**Authors:** Audrey Elford, Alison C Spence, Amy Wakem, Margaret Rozman, Karen J Campbell, Penelope Love

**Affiliations:** 1 Institute for Physical Activity and Nutrition (IPAN), School of Exercise and Nutrition Sciences (SENS), Deakin University, Burwood, VIC, Australia; 2 Nutrition Australia, Melbourne, VIC, Australia

**Keywords:** Early childhood, Childcare, Food waste, Food cost, Environmental sustainability

## Abstract

**Objective:**

To describe environmentally sustainable (ES) and healthy food provision practices in childcare services in Victoria, Australia.

**Design:**

Cross-sectional study.

**Setting:**

Childcare services providing food onsite.

**Participants:**

Staff completed an online survey that explored ES food provision practices including purchasing seasonal/local food, food waste awareness/management, and food cost/child/d. A purposively sampled subgroup conducted weighed audits to determine compliance with guidelines and total waste, serving waste (prepared, not served) and plate waste.

**Results:**

Survey results found 8 % of services (*n* 129) had previously conducted food waste audits. Service audits (*n* 12) found 27 % total food waste (range: 9 % - 64 %). Statistically significant differences in plate waste were found between services who had previously conducted food waste audits (7 %) and those who had not (17 %) (*P* = 0·04). The most common ES practice was ‘providing seasonal food’; the least common was ‘maintaining a compost system’ and ‘less packaged foods’. Most services (95 %) purchased foods from supermarkets with 23 % purchasing from farmers’ markets. This was statistically lower for regional/rural services (8 %), compared to metropolitan services (27 %) (*P* = 0·04). Twenty-seven per cent of services spent AUD2·50 or less per child per day on food. Only one audited service provided a menu compliant with childcare food provision guidelines.

**Conclusions:**

Childcare settings procure and provide large volumes of food; however, food waste awareness appears limited, and environmentally sustainable food procurement practices may be less affordable and difficult to achieve. Understanding the impact of food waste awareness on food waste practices and food costs across time merits further research.

Food has the potential to not only optimise human health but also the health of the planet; however, our current trajectories threaten both^(1)^. It is therefore imperative that governments, organisations and individuals put efforts towards the sustainability of human and planetary health^(1,2)^. Eating preferences and habits are formed in infancy and early childhood and track into adolescence and adulthood^(3)^. Early childhood education and care (‘childcare’) settings are potentially important locations for public health nutrition interventions targeting early childhood due to the high and growing attendance in these settings in high-income countries. For example, attendance in the United States, the United Kingdom and Australia range between 48 % and 64 % of children under the age of 5 years^(4–6)^. In Australia alone, there were 8669 approved centre-based childcare services in 2021, with 816 070 children attending for an average of 31·4 h per week^(6)^.

The American Society of Nutrition recommend that children receive 50 % of their daily intake whilst in childcare^(7)^; however, evidence of food provision in childcare settings in high-income countries shows under-provision of vegetables, whole grains and meat/alternatives and overprovision of foods high in sugar, fat and salt, and highly processed foods^(8,9)^. Food provision research in the childcare setting also indicated potential differences based on service characteristics, with some research indicating that not-for-profit services provided higher quality menus, and services located in regional areas reporting lower skills to plan healthy menus^(8,10)^.

In addition to the potential poorer health outcomes due to current food provision practices in childcare, the foods provided are also less likely to be environmentally sustainable given the high use of ultra-processed and packaged foods^(1,11)^. The Food and Agriculture Organisation outlines that healthy and sustainable diets include foods that are minimally processed, contain whole grains and a variety of fruits and vegetables, and protein sources with limited red meat, have minimal packaging, reduce food loss and waste, and support locally and ethically sourced foods^(11)^.

There is currently limited research within childcare settings regarding environmentally sustainable food provision. Two studies (in Finland and the United States) assessed food waste in childcare and found total food waste to be 20·8 % and 43 % of food served, respectively^(12,13)^. The Finnish study assessed food waste in several different settings including childcare, restaurants and schools and found that of all these settings, childcare had the highest levels of food waste^(13)^. This study however did not explore specific food waste practices within childcare. The United States (US) study weighed food served and wasted and calculated average consumption of food but did not explore their findings in the context of the environmental impacts of food waste^(12)^. Neither of the studies assessed food waste management practices. In addition, there is currently no research within the childcare setting that has assessed environmentally sustainable food procurement practices or environmentally sustainable food provision in the context of menu compliance to meet child-specific nutrition guidelines. Food cost has been identified as a barrier to the provision of both healthy^(14)^ and sustainable food provision^(15)^ in the childcare setting and is therefore an important consideration in the context of affordable, healthy and sustainable food provision.

The development of interventions and support strategies to encourage food provision that is aligned with meeting nutritional needs, environmentally sustainable targets and food budgets requires an understanding of the current practices in place, as well as differences in practices by service characteristics. Therefore, this study aimed to describe environmentally sustainable and healthy food provision practices (including food waste, food costs and service characteristics) in childcare services that provide food onsite in the state of Victoria, Australia.

## Methods

The study utilised an online survey followed by a 1-day audit capturing food served and wasted in childcare services in Victoria, Australia. Data were collected between 2021 and 2022.

### Recruitment

Eligible services were centre-based care (also known as long day care), that operated at least 8 h per day, 48 weeks per annum, and prepared food onsite. Due to these criteria, family childcare, preschool and kindergartens were excluded from our study. Recruitment was undertaken at multiple time points to accommodate for the impact of COVID pandemic lockdowns across Victoria. At the time of recruitment, no publicly available data were available to confirm onsite food provision; therefore, all childcare services on the Victorian Childcare website^(16)^ (*n* 1082) were invited via email sent to the service director or via the social media platform of the Victorian Department of Health’s Healthy Eating Advisory Service^(17)^. The email was sent from a dedicated email address unique to this study, the ‘Deakin Long Day Care study’. Services were invited to participate in (i) an online survey or (ii) an online survey plus a food waste audit and provided organisational consent via a REDCap online consent form. Up to two follow-up emails were sent. Once organisational consent was provided, the REDCap survey link was forwarded to the service staff, who also individually consented via REDcap. For food waste audits, purposive sampling of services that agreed to participate was stratified by service management type (for-profit/not-for-profit), service location (metropolitan/regional) and socioeconomic status. Services were then contacted via email, followed by up to two phone calls, to provide consent to the audits. As incentives for survey completions, participating staff received AUD20 shopping vouchers, and for audit completions, participating services received AUD100 shopping voucher and an illustrated book encouraging fruit and vegetable consumption.

### Data collection

#### Survey measures

##### Service characteristics

The online survey captured service location (metropolitan/regional) and postcode data. Service name, service location and postcode data were used to verify service management type (for-profit/not-for-profit) and socioeconomic position against the 2022 Australian Children’s Education & Care Quality Authority database^(18)^. Socioeconomic position was categorised according to the socioeconomic indexes for areas (SEIFA) into tertiles: Low SEIFA (decile 1–3), Medium SEIFA (decile 4–7), High SEIFA (decile 8–10)^(10,19)^. To ensure sufficient power for analysis, low and medium tertiles were collapsed to create two groups: Low-Medium SEIFA (1–7) and High SEIFA (8–10).

##### Environmental sustainability (food related)

In the absence of a validated measure for environmentally sustainable food provision in childcare, the online survey included purpose-developed questions on food procurement and food cost^(8,20)^, guided by the Food and Agriculture Organisation of the United Nations guiding principles for sustainable healthy diets^(11)^ and the Early Learning Sustainability audit^(21)^. The online survey was tested for face validity by nutrition and early education and care research experts (*n* 6), and adaptions were made. It was further pilot-tested by nutrition students (*n* 6), one of whom had previously worked as a childcare staff member.

The online survey contained thirteen predefined questions including Yes/No questions (for example: ‘Does your centre-based policy include environmental sustainability guidelines such as food waste management?’); Likert scale questions (Never, Sometimes, Most of the time, Always, for example: ‘When planning the menu, we focus on reduced packaged foods’); and a single scale question (1 – not important to 100 – extremely important: ‘When planning the menu, how important is environmental sustainability?’). Average food cost/child/day was based on a question in previous surveys in this setting^(20)^ and stated ‘Please indicate your average daily food cost per child – for example $3/child/d, with an additional explanatory note (see online Supplementary File 1). This question required a dollar amount to be entered. Two short answer questions to further explain previous answers (e.g. ‘Please describe the outcomes of your last food waste audit’). (Full online survey available in online Supplementary File 1)

#### Weighed food waste audits

##### Protocol development

**Fig. 1:**
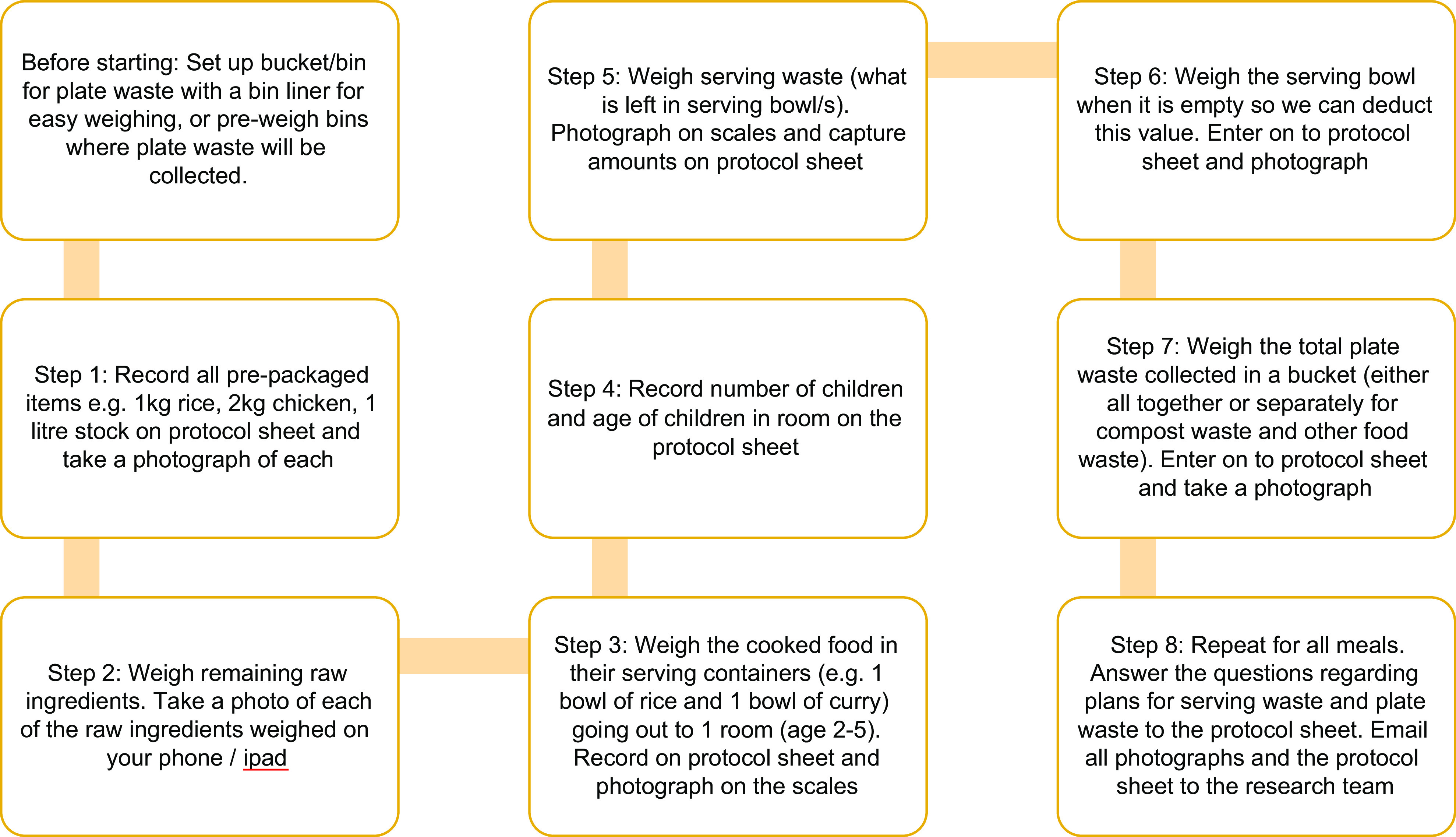
1-day weighed food waste audit protocol (self-measurement)

The planned protocol for measuring food served and wasted in this study was an onsite weighed measurement by trained nutrition researchers with food waste divided into food group components. However, ongoing COVID pandemic lockdowns, and prohibition of entering childcare services when lockdowns lifted, lead to a change in methodology to self-measurement. The protocol was based upon previously developed methodology used to measure food served and wasted at service level in Australian childcare services^(22)^, adjusted for the current study to report total food waste, serving waste (prepared but not served) and plate waste (served but not eaten). The self-measurement protocol was first pilot-tested and refined in two childcare services (not included in the reported data) and provided evidence of suitability for our research purposes. Figure 1 outlines the protocol. A systematic review on food waste audit methods in hospitals lead to the development of a consensus pathway food waste audit tool, which suggested that a 1-day weighed food waste audit can provide a useful baseline measurement or ‘snapshot’ of food waste levels, and this was deemed more practical in the childcare setting rather than the other option and ‘gold standard’ suggestion of 2-week audits^(23,24)^.

##### Food waste audit measures

**Fig. 2:**
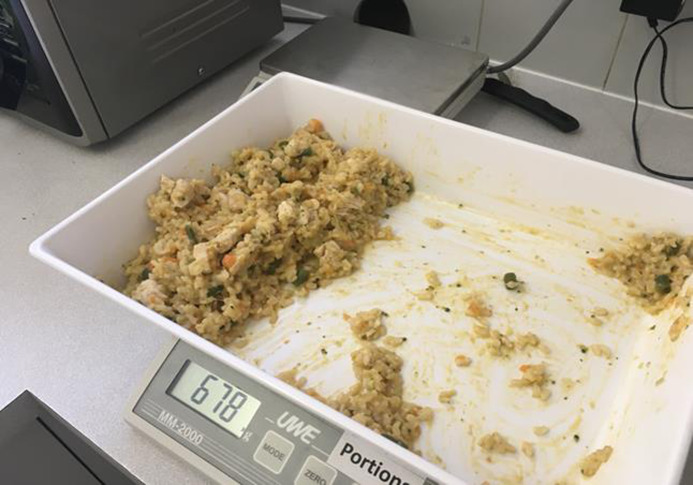
An example photograph of serving waste (food prepared but not served)

Each service was sent a calibrated commercial scale via courier with instructions to check calibration prior to the audit and a phone call prior to the audit day to confirm the understanding of the protocol. Service directors/cooks weighed all food/drink items, completed the protocol template, and photographed items being weighed on the scale using a mobile phone or other digital device (e.g. tablet). This was done at food preparation stage for the entire childcare service and at food serving stage for one 2- to 5-year-old room, chosen by service director discretion for practical reasons. Food prepared but not served to children (serving waste) for the 2- to 5-year-old room was then weighed, recorded and photographed. Food plated and served to children but not eaten (plate waste) was scraped into a bucket by children/educators and weighed, recorded and photographed. The completed protocol templates and all photographs were emailed to the research team, where verification checks of recorded weights on protocol templates were made against photographs of weighed items (see Fig. 2).

##### Additional measures

The protocol template also captured data regarding number of children served and their age range, as well as what services planned to do with the serving waste and plate waste.

### Data analysis

All survey data were downloaded by AE and cross-checked by PL and AS. The data analysis plan was developed by AE and checked by PL, AS, KC and the faculty statistician. STATA do files were created by AE and cross-checked by AS.

#### Surveys

Survey data were downloaded, and tabulations and summaries were used to describe data using Stata 17 statistical software^(25)^. Variables were divided into groups to determine whether there were differences based on service characteristics (for-profit/not-for-profit, metropolitan/regional and low-medium socioeconomic status (SEIFA)/high socioeconomic status). Where variables were continuous, independent *t* tests were used to determine differences in mean values by service type, location or socioeconomic position. One-way ANOVA tests were used when one variable was continuous and the other categorical, and chi-square tests where both variables were categorical. For Likert scale items, a score out of four was created (based on (1) never, (2) sometimes, (3) most of the time, (4) always); therefore, a score of four indicated that this was a practice that was consistently implemented. Mann–Whitney U test was used to determine differences in means of these scores. To manage missing data, available case analysis was utilised, and the number of observations reported. Statistical significance was set at *P* ≤ 0·05.

#### Audits

Healthfulness of the menu was assessed by analysing food group compliance to the Victorian Menu planning guidelines for childcare services^(26)^ using the web-based menu assessment tool, FoodChecker^(27)^. To calculate serving waste (prepared but not served), plate waste (served but not eaten) and total waste (serving and plate waste) percentages, food waste data were entered into a coded Excel spreadsheet. For the twelve services who participated in audits, quantitative survey responses and audit results were combined and analysed using Stata 17 statistical software^(25)^ Audit results were stratified based on survey answers relating to whether they had conducted a food waste audit in the past and whether they included environmental sustainability in their centre-based policy. Summaries and tabulations were used to describe food waste data. Mann–Whitney U tests were used to calculate whether there were statistically significant differences in food waste between services who had conducted food waste audits previously or not and services with centre-based policies that included food environmental sustainability *v*. not. For the audits, complete case analysis was utilised.

## Results

### Survey results (*n* 129 services)

#### Participant descriptions

Data were collected from 129 Victorian childcare services providing food onsite, (12 % response rate); however, currently there are no published data on the number of centres in Victoria that provide food onsite. Participants included service directors (*n* 112) and cooks (*n* 17), of whom 43 % had previously received nutrition training. Participating services were mostly from metropolitan areas (*n* 104) and ‘for-profit’ (*n* 76), with representation across low (25 %), medium (46 %) and high (29 %) socioeconomic position (SEIFA). (Table 1)

**Table 1:** Environmentally sustainable food procurement, policy and costs (by service location, type and socioeconomic position)

Variables	Total (*n* 129)		Metro (*n* 104)		Regional (*n* 25)		*P*-value	For Profit (*n* 76)		Not-for-profit (*n* 53)		*P*-value	Low-Medium SEIFA† (*n* 91)		High SEIFA† (*n* 38)		*P*-value
Received nutrition training																	
Yes	43 %	34–51 %	40 %	31–50 %	52 %	33–71 %	0·29	39 %	29–51 %	47 %	34–61 %	0·38	43 %	33–53 %	42 %	28–58 %	0·94
Food cost/child/day																	
Mean‡	$3·80		$3·85		$3·65		0·52	$3·87		$3·71		0·69	$3·74		$3·98		0·51
95 % CI	3·50–4·20		3·20–4·30		3·10–4·20			3·43–4·31		3·17–4·26			3·30–4·20		3·50–4·50		
≤ $2·50/child/d	27 %	20–36 %	28 %	20–37 %	24 %	11–44 %		22 %	14–33 %	34 %	22–48 %		29 %	20–39 %	24 %	13–40 %	
Above $2·50/child/d	73 %	64–79 %	72 %	63–80 %	76 %	56–89 %	0·69	78 %	67–87 %	66 %	52–78 %	0·15	71 %	61–80 %	76 %	60–87 %	0·57
Purchasing food from:																	
Supermarket																	
Weekly/fortnightly/monthly	95 %	89–97 %	93 %	86–97 %	100 %		0·18	95 %	87–98 %	94 %	84–98 %	0·92	97 %	90–99 %	89 %	75–96 %	0·09
Bulk food store																	
Weekly/fortnightly/monthly	42 %	34–51 %	44 %	35–54 %	32 %	17–52 %	0·26	42 %	31–54 %	42 %	29–55 %	0·95	43 %	33–53 %	39 %	25–56 %	0·72
Delivery service																	
Weekly/fortnightly/monthly	48 %	40–57 %	50 %	40–60 %	40 %	23–60 %	0·39	49 %	38–60 %	47 %	34–61 %	0·86	49 %	39–60 %	45 %	30–61 %	0·63
Farmers market																	
Weekly/fortnightly/monthly	23 %	17–31 %	27 %	19–36 %	8 %	2–27 %	0·04*	20 %	12–30 %	28 %	18–42	0·26	22 %	15–32 %	26 %	15–43 %	0·59
Local butcher/fruit/vege shop																	
Weekly/fortnightly/monthly	67 %	58–74 %	66 %	57–74 %	68 %	48–83 %	0·88	67 %	56–77 %	66 %	52–78 %	0·89	69 %	59–78 %	61 %	44–74 %	0·34
Food from own veg garden§																	
Weekly/fortnightly/monthly	54 %	45–64 %	60 %	49–70 %	38 %	20–60 %	0·1	53 %	41–66 %	57 %	43–71 %	0·65	56 %	45–67 %	52 %	33–70 %	0·68
Environmental sustainability included in policy:																	
Yes	67 %	59–75 %	66 %	57–75 %	72 %	52–86 %	0·59	68 %	57–78 %	66 %	52–78 %	0·78	67 %	57–76 %	68 %	52–81 %	0·88
Importance of environmental sustainability when planning a menu (range 1–100)																	
Mean	65		64		69		0·33	64		65		0·77	65		64		0·81
95 % CI	60–69		59–68		61–76			58–70		60–71			60–70		57–70		

* Statistically significant – statistical significance was calculated with independent *t* test when both variables were continuous, one-way ANOVA when one variable was categorical and the other continuous, and chi-square tests where both variables were categorical.

† SEIFA – Socioeconomic Indexes for areas.

‡ Based on *n* 120 observations.

§ Based on *n* 105 observations.

#### Food procurement

Mean food cost per child per day was AUD3·80 with almost a third of services (27 %) spending AUD2·50 or less. Services mainly purchased foods from supermarkets (95 %), whilst also purchasing from bulk food stores (42 %), local delivery services (48 %), local butchers and fruit/vegetable stores (67 %). Fifty-four per cent of services reported that they regularly used food from their own vegetable garden. Twenty-three per cent of services stated that they bought food from farmers or farmers’ markets, and this was less likely in regional (compared to metropolitan) services (*P* = 0·04). There were no other statistically significant differences by service management type or socioeconomic position.

#### Environmental sustainability importance

About two-thirds of services (67 %) referred to environmental sustainability, including food waste in their centre-based policy. The average value assigned to the importance of environmental sustainability when planning the menu was 65/100, with 32 % of services (*n* 41) assigning a value of below 50/100, indicating low importance of environmental sustainability when planning the menu. There were no differences found by service management type, location, or socioeconomic position.

#### Environmentally sustainable practices

Mean scores for environmentally sustainable practices (0 – never, 4 – always) indicated lowest scores were for ‘purchasing less packaged foods’ (mean score of 2·7/4, with 43 % never or sometimes focusing on this practice) and ‘maintaining a compost system/worm farm’ (mean score of 2·9/4, with 35 % never or sometimes engaging in this practice). Highest scores were for ‘unprocessed foods on the menu’ (3·5/4), ‘seasonal food on the menu’ (3·4/4) and ‘plant-based protein on the menu’ (3·4/4). There were no statistically significant differences by service management type, location or socioeconomic position.

#### Past food waste audits

Based on the surveys, ten services (8 %) had previously conducted food waste audits. Reported consequences of these food waste audits highlighted three outcomes: (a) introducing food waste managing practices, for example, composting (*n* 3), (b) updating the menu to reduce waste (*n* 4), (c) no change indicating acceptable food waste (*n* 3).

### Audit results (*n* 12 services)

#### Description of services participating in audits

Sixteen services consented to participate in audits, and of these, twelve services conducted audits, one service provided missing data and was excluded, and three services withdrew due to staff shortages with COVID pandemic lockdowns at the time. Participating services were equally divided between metropolitan (*n* 6) and regional areas (*n* 6), with representation across low (25 %), medium (58 %) and high (17 %) socioeconomic position. Two-thirds of the services were ‘for-profit’ (*n* 8). Of the twelve services that participated in the 1-day food waste audits, 33 % (*n* 4) had conducted food waste audits in the past, compared to 8 % in the wider online survey.

#### Food waste

Total food waste ranged between 9 % and 64 % (mean 27 %). Serving waste (prepared, not served) ranged between 0–50 % (mean 15 %) and was slightly higher than plate waste (served, not eaten) which ranged between 23–32 % (mean 13 %). There was a trend towards lower food waste across all three categories (total food waste, serving waste and plate waste) in services who had conducted food waste audits in the past, with a statistically significant difference in plate waste between those who had not conducted a food waste audit in the past (17 %) and those who had conducted a food waste audit in the past (7 %) (*P* = 0·04). As outlined in Table 2, there were no statistically significant differences in food waste between services with a policy that included environmental sustainability and those that did not.

**Table 2:** Food waste audit results (stratified by food waste awareness and environmental sustainability included in policy)

Waste type	Mean (%)	Food waste audit conducted in past?		*P*-value	Environmental sustainability included in centre-based policy?		*P*-value
		Yes (*n* 4)	No (*n* 8)		Yes (*n* 8)	No (*n* 4)	
Total food waste	27 %	18 %	34 %	0·16	25 %	32 %	0·97
Total serving waste†	15 %	12 %	18 %	0·32	12 %	23 %	0·38
Total plate waste‡	13 %	7 %	17 %	0·04*	14 %	23 %	0·62

Mann–Whitney U tests were used to calculate differences in percentages.

* Statistically significant.

† Serving waste = food prepared but not plated/served.

‡ Plate waste = food served/plated but not eaten.

#### Plans for food waste

Plate waste (served, not eaten) was mainly used in compost bins/worm farms or to feed animals (*n* 10), with some services discarding plate waste in regular bins (*n* 2). Serving waste (prepared, not served) was either used for meals/snacks the next day (*n* 4), offered to staff to consume during their lunch (*n* 4), used in compost bins (*n* 2), discarded in regular bins (*n* 1) or packaged into servings for parents (*n* 1).

#### Menu compliance (to assess healthfulness of the menu)

Based on the web-based menu assessment tool FoodChecker, one of the twelve audited services had a compliant menu that provided the required food groups per day as per the Victorian menu planning guidelines^(26)^. Whilst the menu was compliant in terms of providing all food groups, there was overprovision of recommended quantities for all food groups, especially meat/alternatives with almost 4 times the recommended amount provided. This service had an average total waste of 21 % (serving waste 10 %; plate waste 11 %).

The menus of the remaining eleven services under-provided at least one food group. Services mostly under-provided grains (*n* 7) and dairy/alternatives (*n* 6). Two-thirds of services (*n* 7) provided highly processed foods, high in fat, sodium, or sugar and not recommended for children in childcare. Some food groups, mostly fruit (*n* 6) and vegetables (*n* 5), were over-provided. Average waste for services with non-compliant menus (*n* 11) was 29 % (serving waste 16 %; plate waste 13 %).

## Discussion

### Food waste

Our study survey found that there was low food waste awareness in services, with only 8 % having conducted food waste audits in the past. In addition, our weighed food waste audits indicated that services with higher food waste awareness (had conducted a food waste audit in the past) had an average of 41 % less plate waste. Our audits were conducted in a small number of services, and these findings need to be confirmed in larger studies. However, similar trends have been reported in related settings. A recent study in Swedish school canteens tested four interventions to reduce food waste: tasting spoons (for students to taste the dish before serving themselves), a food waste awareness campaign, plate waste tracker and a forecasting tool^(28)^. Whilst all interventions led to food waste reduction, the food waste awareness campaign had the largest impact on plate waste, reducing it by 35 %^(28)^. Similarly, a pre- to post-intervention study on a food waste awareness campaign at the University of Lisbon indicated that plate waste was reduced by 15 % after the awareness campaign^(29)^. That study also found that after the pre-intervention food waste measures, the canteen menus were changed to eliminate high plate waste menu items, which the authors suggest may have contributed to the reduction in plate waste^(29)^. This was also a finding in our study, where 40 % of services who had conducted food waste audits in the past made changes to their menus to reduce the food waste. Future larger studies could investigate whether conducting food waste audits in this setting may be an effective food waste reduction intervention on the premise of catalysing actions and strategies to reduce food waste.

Overall, our study audits found a mean total food waste of 27 %; however, it is important to recognise that a third of the services that participated in our audits had conducted food waste audits in the past, compared to 8 % in the larger survey cohort. Given this higher percentage of food waste awareness, this may indicate that the food waste levels reported in our audits are a best-case scenario rather than usual practice. Despite this, total food waste levels in our study were higher than those reported in a Finnish study, which found that 20·8 % of food prepared in childcare services (*n* 13) was wasted, of which serving waste constituted 13·2 % and plate waste 2·9 %^(13)^. The differentiation of total food waste into serving waste and plate waste, as done in our study and others, is important, as where food waste occurs lends itself to different strategies for improvement. For instance, food service staff may perceive serving waste to be related to overprovision and difficulties in prediction of how much food to prepare^(13,29)^ and plate waste to be related to poor acceptability of the recipes^(13)^. To inform appropriate food waste reduction actions and strategies within the childcare setting, future food waste studies should make the distinction between serving waste and plate waste and explore perceptions of childcare staff regarding the underlying reasons behind these two food waste components.

### Food waste mitigation strategies

A third of services in our study reported not regularly engaging in unavoidable waste mitigation strategies, such as in maintaining a compost bin or worm farm. These strategies reduce the volume of food waste that goes to landfill and can increase overall environmental sustainability awareness in those who participate in this practice^(30,31)^. Little is reported on waste mitigation in the early education setting. A Canadian study exploring hospital service staff’s beliefs, attitudes and behaviours regarding environmentally sustainable food practices^(32)^ suggested 88 % of participants believed food waste composting to be an important practice, but only 43 % engaged in composting practices. The authors suggested that research investigating barriers and enablers to the implementation of environmentally sustainable food practices was needed^(32)^. Future investigation on the barriers and facilitators of the establishment and maintenance of onsite composting facilities within the childcare setting could inform appropriate support to adopt this strategy.

### Food waste in the context of healthy food provision (menu compliance)

Although audited services with non-compliant menus (*n* 11) under-provided for at least one food group, they still reported food waste ranging from 9–64 %. Food waste was also reported for the audited service with a compliant menu (*n* 1), which was over-provided for all food groups. Whilst these findings are from a small sample, it highlights the importance of including food waste as a consideration when assessing menu compliance, as simply increasing food quantities to provide more compliant menus may lead to increased food waste. This was found in a US study where preschools with compliant menus were found to waste 43 % of the food served, mainly consisting of fruit (38 %) and vegetables (61 %)^(12)^. In another US study of preschool services participating in the Child and Adult Care Food Program (a federal government programme that provides reimbursements to childcare services for serving menus compliant with nutrition guidelines)^(33)^ found that whilst menus provided were mostly compliant with guidelines, children’s intakes were not, with the difference leading to food waste^(34)^. A narrative review on the link between nutrition, food waste and environmental sustainability suggested that a focus on reducing food waste could potentially lead to poorer diet quality, and the authors pointed to the clear need for nutritional quality and food waste messaging to occur simultaneously^(35)^. Current practice guidelines to childcare focus on the healthfulness of the food, but not the environmental sustainability, pointing to a need for policy and practice guidelines to reflect both.

### Feeding practices aligned with healthy and sustainable food provision

Another factor to consider is not only what is served but also how it is served, namely feeding practices where food is pre-plated or if children are encouraged to self-serve. The link between feeding practices, consumption patterns and food waste has recently been identified in an Australian initiative to increase fruit and vegetable acceptance and consumption in childcare settings, named VegKit^(36)^. VegKit targets childcare service cooks (through online training on menu planning and an online menu assessment tool) and educators (through online training on role modelling and encouragement of fruit and vegetable consumption). Preliminary evaluation of VegKit found that the intervention doubled fruit and vegetable intake whilst reducing food waste, with staff training considered an important component for successful provision of healthy and sustainable food^(36)^. Our study survey found that less than half of the respondents had previous training in nutrition, similar to another recent study in Australia which found that 43 % of staff members responsible for planning meals had received training in nutrition^(17)^. This indicates a need for childcare staff training and support regarding the planning, procurement, preparation and provision of healthy and sustainable menus, as well as feeding practices that enhance consumption of the food served.

### Food budgets and sustainable food procurement

A possible further challenge to the successful implementation of healthy and environmentally sustainable food provision in childcare settings is the food budget. We found an average food cost per child per day of AUD3·80, with a third of services having a food budget of AUD2·50 or less per child per day. No differences were found by socioeconomic position of the service, which may indicate that this issue is being experienced widely. It is important to mention that a limitation of this study is the estimation of food costs by staff, rather than calculation of actual food costs through receipts or costing by food items; therefore, the findings need to be interpreted in the light of this limitation. Despite this, a study conducted in Western Australia two years prior to our study used food items provided to menus to cost actual menus to the food prices at local supermarkets^(37)^. They found similar results to our study, in that the average food cost per child per day was AUD2·00 and this was not consistent with providing a compliant menu. That study reported that increasing the food budget to AUD2·50 per child per day could ensure that four out of the five food groups were compliant with menu guidelines^(37)^. Whilst that study did not report what a fully compliant menu would cost, it is unlikely that a healthy menu on a budget of AUD2·50 is feasible in today’s climate of rising food prices. Additionally, our study did not report on the costs of food waste, which could be a motivating factor in future interventions and warrants further investigation.

Sustainable food procurement might be more costly, as outlined by an Australian study that found that, depending on locality, a healthy and sustainable food basket was 4 % - 30 % more expensive compared with a typical food basket^(38)^. In the US, sustainable food procurement in the form of ‘farm to childcare’ programmes found the main barriers to local purchasing, including farmers’ markets were the costs and difficulties in finding suppliers or farmers^(15)^. This is similar to the findings in our study which indicated that farmers’ markets are not often utilised for food procurement, particularly in regional areas where access might be an issue. The use of farmers’ markets in our study was however higher than a US food procurement study in 2018, which reported that 1 % of services procured food from farmers’ markets compared to 23 % in our study^(39)^ A possible explanation for this is that our study was conducted during and shortly after the COVID pandemic, where, due to disruptions in food supply chains, there was an increase in purchasing from local food systems across high-income countries^(40)^. Future food procurement studies in this setting should confirm that this trend in purchasing more local food compared to previous studies, particularly given the barrier of food costs and rise in food prices. Encouragingly, around half of the services in our study used some food from their edible gardens in their menus. Edible gardens are a popular choice in childcare services, with a New Zealand study reporting that nearly all (89·5 %) of their 257 services had an edible garden onsite^(41)^, and a UK study indicating that most childcare services (81 %) were interested in growing their own garden; however, the UK study indicated that space, time and expertise were barriers^(42)^. Additionally, it may take more time to plan a menu responsive to seasonal food availability, procure sustainably and prepare more plant- and whole grain-based meals, further contributing to resourcing and cost challenges of sustainable meals^(43)^. These challenges, in combination with insufficient resources and training as well as restrictive budgets, likely make healthy and sustainable food provision in childcare difficult to achieve, indicating a need for policy action to support this sector.

### Strengths and limitations

Our study used a multi-method approach, combining survey data with good reach and detailed measured audits. The methodology combined the gold standard (weighed foods) with photography to reduce human error due to self-measurement. This self-measurement audit tool enabled the continuation of the research during COVID-19 and should be explored as a future opportunity for larger studies, reducing the need for researchers to collect this data within childcare facilities. Self-measurement could also have the added benefit of increasing food waste awareness in the staff conducting the audits. The measurement of menu compliance alongside food waste ensured that we were able to consider both healthfulness and environmental sustainability of the food provided. Finally, our research sets the stage for future triple-duty action research and interventions in childcare settings, which has been found to be more effective than addressing each component in isolation^(44)^.

Our study has several limitations. As a cross-sectional study, no causality can be inferred, and future controlled intervention studies are recommended. The COVID pandemic impacted survey response rates and, combined with the small sample participating in food waste audits, warrants larger studies to confirm the findings. In addition, with a third of the services in our audits having conducted food waste audits in the past compared to 8 % in the wider survey, it is likely that selection bias occurred in our audit sample, potentially skewing the food waste results towards more of a best-case scenario rather than usual practice. Whilst the self-measurement of food waste is potentially a more feasible approach to measure food waste in services that are physically dispersed, and this method enabled data collection during and after COVID pandemic lockdowns, the method is a limitation in our study as service staff may not have collected accurate data. Therefore, the self-measurement audit tool might be a future opportunity for larger studies; however, this methodology will need to be validated. Due to practical reasons for managing participant burden, the self-measurement methodology also limited the opportunity to calculate levels of food waste according to specific food groups. To reduce burden on services, we conducted a 1-day snapshot in one 2–5-year-old room, which may not reflect overall long-term food waste patterns across the entire service. Our survey did not capture the maximum number of children attending per day which did not allow us to stratify our findings by actual size of service. Future studies could potentially investigate whether smaller services are more likely to engage in environmentally sustainable practices compared to larger services. Finally, as there are currently no publicly available data on the number of centres that provide food onsite nor overall Victorian childcare centre characteristics, we were unable to compare our sample to the target sample for representativeness.

### Conclusions

Early childhood provides a window of opportunity to cultivate food habits that can influence both human and planetary health, and high attendance in childcare settings suggests that targeting healthy and environmentally sustainable food provision could have an impact at scale. Our study found that whilst some environmentally sustainable food provision practices are in place, there appears to be limited food waste awareness and low engagement in environmentally sustainable food waste management practices within childcare services. The positive impact of food waste awareness on food waste reduction practices in the childcare setting warrants further research. Our study highlighted insufficient nutrition training and restrictive food budgets as additional potential barriers to the incorporation of environmentally sustainable food provision and practices. The intersection of healthy, environmentally sustainable and affordable food provision remains under-researched and is likely difficult to achieve. The findings in this study indicate that the childcare sector requires strong policy action and practice guidelines to support environmentally sustainable, healthy and affordable food provision.

## Supplementary material

For supplementary material accompanying this paper visit https://doi.org/10.1017/S1368980023001908. click here to view supplementary material
