# Supplementary material for: Environmental sustainability and food provision in the early childhood and education setting
Source: Public Health Nutr. 2023 Oct 9;26(11):2200–10. doi: 10.1017/S1368980023001908 (PMC10641621; doi:10.1017/S1368980023001908)
Supplement: Supplementary file 1 [file S1368980023001908sup001.pdf]

Environmentally sustainable food in LDC survey

Deakin Long Day Care Study

Welcome to the Environmentally sustainable food in LDC survey

You are eligible to participate in this study if you are the director of a childcare centre that:

a) operates for at least 8 hours per day AND

b) provides food onsite (morning and afternoon tea and a main midday meal)

Consent

This 5-10 minute survey will ask you about the environmentally sustainable food practices in your childcare centre. If you need to stop the survey before its completion, a link will be emailed to you to enable you to complete it later.

More information about the study is available in the attached Plain Language Statement. If you have any questions, please email the research team at ldcstudy@deakin.edu.au.

[Attachment: "Env Director survey PLS and consent form .pdf"]

Name of Long day care centre

____

Postcode of Long day care centre

____

By selecting YES I am confirming that:

a) I have read and understand the attached Plain Language Statement.
b) I freely agree to participate according to the conditions in the Plain Language Statement.
c) I have downloaded a copy of the Plain Language Statement and Consent Form to keep.
d) I understand that the researchers have agreed not to reveal my identity and personal details, including where information about this project is published, or presented in any public form.

I give consent to participate in this survey.

◯ Yes
◯ No

Your First and Last name

Note: this information will only be used to contact you if you won the draw for the voucher and not for any other purposes

____

Organisational Email Address

Note: Please provide a valid work email address. This email will only be used to send you a reminder if you are unable to complete this survey in one attempt, and to send you your gift voucher after completing this survey if you won the draw

____

What is your country of birth?

____

Do you speak a language other than English?

◯ Yes
◯ No

Please specify

____

Section A: About your role

A1. How long have you worked in your current role as Director?	◯ Less than 12 months<br>◯ 1-2 years<br>◯ 2-3 years<br>◯ 3-4 years<br>◯ Over 4 years
A2. What is your highest level of educational attainment?	◯ University Degree<br>◯ Trade, Apprenticeship, Diploma, certificate<br>◯ Year 12<br>◯ Year 11 or below
A3. Have you had any training in nutrition to support you in your role?	◯ Yes<br>◯ No
A3.1. Please list	

Section B: About your centre

B1. Is your centre privately managed or community owned?	◯ Private<br>◯ Community
B2. Total number of enrolments (aged 1-5 years) at your centre	______________________
B3. Please indicate which of the following age groups you have at your centre. Select all that apply.	☐ < 1 year<br>☐ 1 year<br>☐ 2 years<br>☐ 3 years<br>☐ 4 years<br>☐ 5 years<br>(Mark all that apply)
B4. Total number of staff at your Long Day Care Centre (including the Director)	______________________

Section C - Food purchasing, preparation and budget

C1. How often do you purchase food from the following:

	weekly / fortnightly	monthly	never
supermarket	○	○	○
bulk food store (e.g. Costco)	○	○	○
local food delivery service (not supermarket)	○	○	○
farmer's market	○	○	○
local butcher / fruit and vege store	○	○	○
other	○	○	○

C1.1 If you indicated "other" in the previous question, please specify here

C2. Please indicate your average daily food cost per child – for example $3/child/day. ______________________________

Please note: if you quote food cost in any other form (e.g. per week or per day over the span of your whole centre) please clearly state what your amount is referring to.

C3. Who is responsible for the following:
(Please note: you can select multiple options for each so please select all that apply)

	a designated cook / other preparation staff	educators	directors	other
food shopping / food ordering	☐	☐	☐	☐
food budgeting	☐	☐	☐	☐
menu planning	☐	☐	☐	☐
food preparation	☐	☐	☐	☐

You have indicated "other" in the previous section. Please provide details of the staff member responsible for the food provision tasks on the previous question.

C4. Do any of these staff have training in nutrition that includes understanding of dietary guidelines? (note: in this case dietary guidelines are referring to child-specific guidelines on food groups and amounts needed per day)

	yes	no	unsure
staff responsible for food shopping/ ordering	○	○	○
staff responsible for food budgeting	○	○	○
staff responsible for menu planning	○	○	○
staff responsible for food preparation	○	○	○

Section D: Environmentally sustainable food practices at your centre.

D1. Does your centre policy include guidelines on environmental sustainability such as food waste management?

◯ Yes
◯ No

D2. When planning the centre menu, how much importance is placed on environmental sustainability. Please use the slider to change value, where 1 is not very important and 10 is extremely important.

1 5 10

(Place a mark on the scale above)

D3. Is there a garden available at your centre for children to plant/grow items?

◯ Yes
◯ No

D3.1 What is in the garden?

☐ flowers
☐ herbs
☐ fruits
☐ vegetables

D3.2 Of the fruits and vegetables grown, are there enough

◯ for tasting only
◯ to serve as part of meals and snacks

D4. Please answer the following statements regarding environmental sustainability (food related) in your centre.

	Never	Sometimes	Most of the time	Always
The majority of food on our menu is fresh, unprocessed food.	◯	◯	◯	◯
When planning the menu, our centre focuses on seasonal produce.	◯	◯	◯	◯
When planning the menu, our centre focuses on reduced packaged foods.	◯	◯	◯	◯
Our menu regularly features vegetarian meals that contain legumes.	◯	◯	◯	◯
Food scraps during food preparation are collected in the kitchen.	◯	◯	◯	◯
Children are encouraged to scrape bowls and plates into a scraps bowl after meals and snacks.	◯	◯	◯	◯
Our centre maintains a compost system, worm farm, chicken coop, and/or other	◯	◯	◯	◯
Food scraps are either added to worm farms, compost facilities, or provided as food for chickens as appropriate.	◯	◯	◯	◯

D5. Other than using food scraps in compost or worm farms, does your centre have a plan on using food leftovers? ◯ Yes ◯ No

D5.1. Please describe how leftovers are used

____

D6. Has your centre ever done a food waste audit? ◯ Yes ◯ No

D6.1. Please describe the outcomes of your last food waste audit.

____

Thank you so much for your time.

Please press SUBMIT
